# Effective connectivity of the right anterior insula in schizophrenia: The salience network and task-negative to task-positive transition

**DOI:** 10.1016/j.nicl.2020.102377

**Published:** 2020-08-07

**Authors:** Qiang Luo, Baobao Pan, Huaguang Gu, Molly Simmonite, Susan Francis, Peter F. Liddle, Lena Palaniyappan

**Affiliations:** a Institute of Science and Technology for Brain-Inspired Intelligence, MOE-Key Laboratory of Computational Neuroscience and Brain-Inspired Intelligence, State Key Laboratory of Medical Neurobiology and MOE Frontiers Center for Brain Science, Institutes of Brain Science and Human Phenome Institute, Fudan University, Shanghai 200433, China; b School of Aerospace Engineering and Applied Mechanics, Tongji University, Shanghai, China; c Translational Neuroimaging for Mental Health, Division of Psychiatry and Applied Psychology, University of Nottingham, Nottingham, UK; d Sir Peter Mansfield Imaging Centre (SPMIC), School of Physics and Astronomy, University of Nottingham, Nottingham, UK; e Robarts Research Institute & The Brain and Mind Institute, University of Western Ontario, London, Ontario, Canada; f Department of Psychiatry, University of Western Ontario, London, Ontario, Canada; g Lawson Health Research Institute, London, Ontario, Canada

**Keywords:** Salience network, Dorsolateral prefrontal cortex, Working memory, Default mode network, Effective connectivity, Schizophrenia

## Abstract

Triple network dysfunction theory of schizophrenia postulates that the interaction between the default-mode and the fronto-parietal executive network is disrupted by aberrant salience signals from the right anterior insula (rAI). To date, it is not clear how the proposed resting-state disruption translates to task-processing inefficiency in subjects with schizophrenia. Using a contiguous resting and 2-back task performance fMRI paradigm, we quantified the change in effective connectivity that accompanies rest-to-task state transition in 29 clinically stable patients with schizophrenia and 31 matched healthy controls. We found an aberrant task-evoked increase in the influence of the rAI to both executive (Cohen’s *d* = 1.35, *p* = 2.8 × 10^−6^) and default-mode (Cohen’s *d* = 1.22, *p* = 1.5 × 10^−5^) network regions occur in patients when compared to controls. In addition, the effective connectivity from middle occipital gyrus (dorsal visual cortex) to insula is also increased in patients as compared with healthy controls. Aberrant insula to executive network influence is pronounced in patients with more severe negative symptom burden. These findings suggest that control signals from rAI are abnormally elevated and directed towards both task-positive and task-negative brain regions, when task-related demands arise in schizophrenia. This aberrant, undiscriminating surge in salience signalling may disrupt contextually appropriate allocation of resources in the neuronal workspace in patients with schizophrenia.

## Highlights

• Neuroimaging study of task-evoked state shift in schizophrenia.
• Salience signaling failure upon placing task demands in schizophrenic patients.
• Increased task-evoked insular output to the executive network.
• Increased task-evoked visual input to the insula.
• More severe negative symptoms were associated with stronger insula output.

## 1 Introduction

The discovery of large-scale brain networks based on functional neuroimaging (fMRI) has provided a robust framework to study the notion of dysconnectivity that has been suspected to bridge the cellular and clinical aspects of schizophrenia (Friston, 1994, Guo et al., 2020, Northoff, 2015). In this regard, a theory driven model of Salience Network dysfunction postulates that aberrant functional organization of two key large-scale cognitive networks driven by the anterior insula (Menon and Uddin, 2010) may contribute to psychopathology in patients with schizophrenia (Palaniyappan et al., 2012). In a Granger-causal fMRI connectivity study Sridharan and colleagues (2008) first reported the primacy of the salience network (SN; right anterior insular cortex [rAI] and dorsal anterior cingulate cortex) in influencing the default mode network [DMN] (comprising of the ventral medial prefrontal cortex and precuneus) and the central executive network [CEN] (dorsolateral prefrontal cortex and posterior parietal cortex) during task processing in healthy subjects. The DMN has been implicated in self-referential processing, while the SN is considered to attribute salience to relevant stimuli and engage CEN for working memory and higher-level cognitive control (Menon, 2011). Subsequently, several effective connectivity studies using either Granger or dynamic causal models have demonstrated that rAI failed to be negatively correlated with CEN and positively correlated with DMN during resting state in schizophrenia when compared to healthy controls (Manoliu et al., 2013, Moran et al., 2013, Palaniyappan et al., 2013).

Impaired rAI modulation of CEN and DMN observed using resting-state fMRI relates to impaired cognitive task performance across various psychotic disorders (Moran et al., 2013, Palaniyappan et al., 2013, Sheffield et al., 2017). To date, it is not clear how the presence of resting-state dysconnectivity centred on rAI translates to task-processing inefficiency in subjects with schizophrenia. Understanding this mechanism is crucial not only to explain how symptoms of schizophrenia are triggered by certain demands placed on an individual, but also to develop an informed approach to therapeutic brain network modulation. The rAI is considered crucial for ‘proximal salience mapping’, i.e. detecting salient external stimuli and internal mental events and allocating appropriate attentional resources to match processing demands (Palaniyappan and Liddle, 2012). As a causal control node that influences the competing operations of the CEN and the DMN (Goulden et al., 2014, Ham et al., 2013, Sridharan et al., 2008, Supekar and Menon, 2012), rAI facilitates transition from a resting to task-processing brain state when cognitive demands arise, by selectively enhancing one network over the other. Based on prior synthesis (Palaniyappan and Liddle, 2012, Palaniyappan et al., 2013) as well as the emerging body of fMRI studies in the last decade (Dong et al., 2018, Supekar et al., 2019), we hypothesized that the control signals from rAI will be abnormally elevated in a non-selective manner when task-related demands arise in schizophrenia. We expected this aberration to be concentrated around the core DMN and CEN nodes across the brain, consistent with a conditional failure of resource allocation in schizophrenia (Braver et al., 1999, Granholm et al., 1996). To this end, we compared the change in the directed influence from rAI between resting and task-processing brain states observed during a single session of fMRI acquisition in 64 subjects.

Cognitive deficits involving working memory are tightly linked to the domain of negative symptoms characterised by psychomotor poverty and persistent functional disability in schizophrenia. While the mechanism of negative symptoms continue to be elusive, Manoliu and colleagues (2013) used independent component analysis of functional connectivity at rest and reported a strong relationship between increased SN to CEN connectivity and negative symptoms in patients with established schizophrenia. Based on this observation, along with the prevalent computational models of negative symptoms (Deserno et al., 2017, Gold et al., 2012, Limongi et al., 2020) indicating a failure of bottom-up signalling in the hierarchical information processing network, we hypothesised that an abnormal task-related increase in rAI to CEN and DMN signalling may underlie the negative symptoms of schizophrenia.

## 2 Material and methods

### 2.1 Participants

The sample consisted of 32 patients satisfying DSM-IV criteria for schizophrenia (SZ) or schizoaffective disorder and 32 matched healthy controls (HC) reported in our prior studies (Palaniyappan and Liddle, 2014, Palaniyappan et al., 2013). Patients were recruited from the community-based mental health teams (including Early Intervention in Psychosis teams) in Nottinghamshire and Leicestershire, UK. All participants gave their written informed consent to participate in our study after detailed description of the risks and benefits. Any data with a head motion exceeding 3.0 mm or rotation exceeding 3.0° were excluded (one patient). Moreover, one healthy control and two patients had exceptionally low task performance (hit rate <30%) and were excluded. Finally, 29 patients with SZ and 31 HC met all inclusion criteria, and matched in age, gender and handedness (Table 1).

**Table 1:** Demography of patients with schizophrenia and healthy control.

Items	SC (n = 29)	HC (n = 31)	P value	statistics
Gender (F/M)	5/24	9/22	0.2805	χ^2^ (1) = 1.16
Handedness (L/R)	5/24	3/28	0.3891	χ^2^ (1) = 0.74
Age in years (SD)	33.2 (9.2)	33.8 (9.2)	0.8132	T_58_ = 0.24
Hit rate (SD)	73.7 (7.5)	78.5 (5.0)	0.0051	T_58_ = 2.91
Chlorpromazine equivalence in mg (SD)	612.5 (564.3)			
Duration of illness in years (SD)	9.0 (7.0)			
Approximate lifetime exposure in mg (SD)	6.1 × 10^3^ (1.0 × 10^4^)			

### 2.2 Neuroimaging

#### 2.2.1 Data acquisition

As described in our prior reports (Palaniyappan and Liddle, 2014) functional MRI images were acquired on a 3 Tesla Philips Achieva MRI scanner (Philips, Netherlands) during 10 min of rest, with eyes open. We acquired dual-echo, gradient-echo, and echo-planar images (GE-EPI) to enhance sensitivity and reduce susceptibility effects, using an eight-channel SENSE head coil with SENSE factor 2 in anterior-posterior direction, TE1/TE2 25/53 ms, flip angle 85°, 255 × 255 mm field of view, with an in-plane resolution of 3 mm × 3 mm and a slice thickness of 4 mm, and TR of 2500 ms. At each dynamic time point a volume dataset was acquired consisting of 40 contiguous axial slices acquired in descending order. Two hundred and forty time points were acquired during the resting fMRI paradigm. After the resting-state fMRI sequence, a visual n-back session immediately followed.

A magnetization-prepared rapid acquisition gradient echo image with 1 mm isotropic resolution, 256 × 256 × 160 matrix, Repetition Time (TR)/Echo Time (TE) 8.1/3.7 ms, shot interval 3 s, flip angle 8°, SENSE factor 2 was also acquired for each participant for volume registration. Weighted summation of the dual-echo images produced a single set of low-artefact functional images (Posse et al., 1999).

#### 2.2.2 N-back working memory task

We used a visual n-back task with a button press response in two sessions of fMRI recording. Seven task blocks each of 110 s duration were presented in each session. Each task block consisted of 0-back, 1-back, and 2-back conditions of 30 s duration. Each condition presented in a random sequence, with 10 s interval between the conditions. On-screen instructions preceded every condition indicating the type of response required (0-, 1-, or 2-back; 2 s). Each condition included four target and 11 non-target stimuli with a 2s inter-stimulus interval. To ensure adequate task comprehension and performance, all participants performed a practice version of the task outside the scanner prior to scanning. All scanned participants successfully identified in excess of 80% of targets in the practice task. Task sessions immediately followed the resting sessions during the acquisitions (See Fig. S1).

#### 2.2.3 Data preprocessing

fMRI data including resting and engaging task were preprocessed using Statistical Parametric Mapping version 8 (SPM8, www.fil.ion.ucl.ac.uk/spm) and DPABI: a toolbox for Data Processing & Analysis for Brain Imaging (rfmri.org/dpabi) (Yan et al., 2016). For the resting-state data, we removed the first five volumes, and applied slice timing, spatial normalisation, “scrubbing” (using interpolation method of ArtRepair), smoothing (with a 8 mm full width at half maximum isotropic Gaussian kernel), and a band-based filter (0.01–0.08 Hz). Nuisance covariates removed by regression six head motion parameters, global mean signal, white-matter signal, and cerebro-spinal fluid signal was removed by regression in line with our prior work (Palaniyappan et al., 2013).

For task fMRI data, we used an additional task regressor per condition convolved with a hemodynamic response function (with default parameters in SPM8) to control for the false connectivity owing to the common associations with the task design (Cole et al., 2013).

Removal of global signal has been shown to reduce physiological noise, including those arising from motion both at rest (Fox et al., 2009, Hayasaka, 2013, Yan et al., 2013) and during task-fMRI (Liu et al., 2017), though there is no consensus on a single best approach (Umeh et al., 2020). As we intended to compare connectivity status during rest with a cognitive task, wherein the differences in voxel-wise relationship of the non-neuronal global signals could influence the direction of observed changes, we chose to regress out the state-specific global signal before deriving Granger-causal coefficients. See Fig. S2 for a summary of the methods.

### 2.3 Statistical analysis

#### 2.3.1 Seed-based analysis of effective connectivity: rest vs. task

We defined a seed-region as a 6 mm-sphere (centered at MNI: 33, 21,-3) for the right anterior insula [rAI] following our previous study of the SN in SCZ using resting-state fMRI (Palaniyappan et al., 2013). We conducted a seed-based whole-brain analysis using the Granger causal modelling (GCM) (Friston et al., 2013, Seth et al., 2015), that had been successfully applied to indicate the directional influence between brain regions during rest (Hamilton et al., 2011, Luo et al., 2013) and task (Kadosh et al., 2016, Luo et al., 2013, Luo et al., 2017, Wen et al., 2013), to disentangle the influences of the rAI on the other voxels in the brain and those influences in the opposite direction.

For the resting scans, we estimated the path coefficient [PC] from the seed to the whole brain by GCM (Pu et al., 2016). For the task fMRI, only 2-back blocks were used for GCM as cognitive demands are consistently higher at this level (Meule, 2017). As required by GCM, detrending and zero-mean normalization were conducted within each 2-back block. We then calculated the same PC in each 2-back block, and then, averaged across blocks to provide a mean PC for the task. Similar analyses were also performed in the reverse direction i.e. whole-brain-to-seed analysis. Following previous reports (Hamilton et al., 2011, Palaniyappan et al., 2013, Wen et al., 2012), we used one-time lag for the GCM. The code is made available at https://github.com/qluo2018/Resting2TaskShifting.

We estimated the change of the path coefficient [CPC] across all voxels [CPC maps] contrasting resting from task-active state (seed-to-whole brain and vice versa).

#### 2.3.2 Change of path coefficient: patients vs. controls

The estimated CPC maps were compared between patients and controls by two-sample *t-*test after adjustment for the covariates age, gender, and head movement (meanFD). Clusters with significantly different CPC were identified after the updated version (Cox et al., 2017) of Alpha-Sim correction on brain mask (Monte Carlo simulations, implemented in the DPABI) for multiple comparisons with voxel-level *p* value < 0.01 and cluster *p* value <0.05. To demonstrate that the difference in connectivity cannot be simply explained by the difference in brain activations, we also tested confounding effect of group-difference in brain activation (e.g., the seed region) on the findings. To determine whether the identified increase in CPC meant a larger positive effect or a change from negative effect to positive effect, we carried out additional one-sample *t-*test for the significant clusters considering the same set of covariates.

#### 2.3.3 Behavioral and symptom correlation

We averaged the CPCs among all voxels within each significant cluster, and tested the behavioural and symptom associations of the CPC at cluster level. Spearman correlation was used for behavior score (Hit rate of task performance) and three symptom scores (Disorganization, Psychomotor Poverty and Reality Distortion, Table S1), while Pearson’s test was used for the Social and Occupational Functioning Assessment Scale (SOFAS; Association, 1994) and Signs and Symptoms of Psychotic Illness (SSPI; Liddle et al., 2002) total score. Using Spearman’s test, we also related CPC values from each cluster to current dose of antipsychotics (using Defined Daily Dose units as well as a proxy measure of cumulative life time exposure to antipsychotics used in prior studies (Methodology, 2003)). To test our prior hypothesis relating Psychomotor Poverty to rAI-to-CEN and rA-to-DMN CPCs, we used a statistical threshold of *p* < 0.025 (two-tailed). For other exploratory correlations, we used an uncorrected threshold (*p* < 0.05).

## 3 Results

### 3.1 Change in path coefficient from rAI

**Fig. 1:**
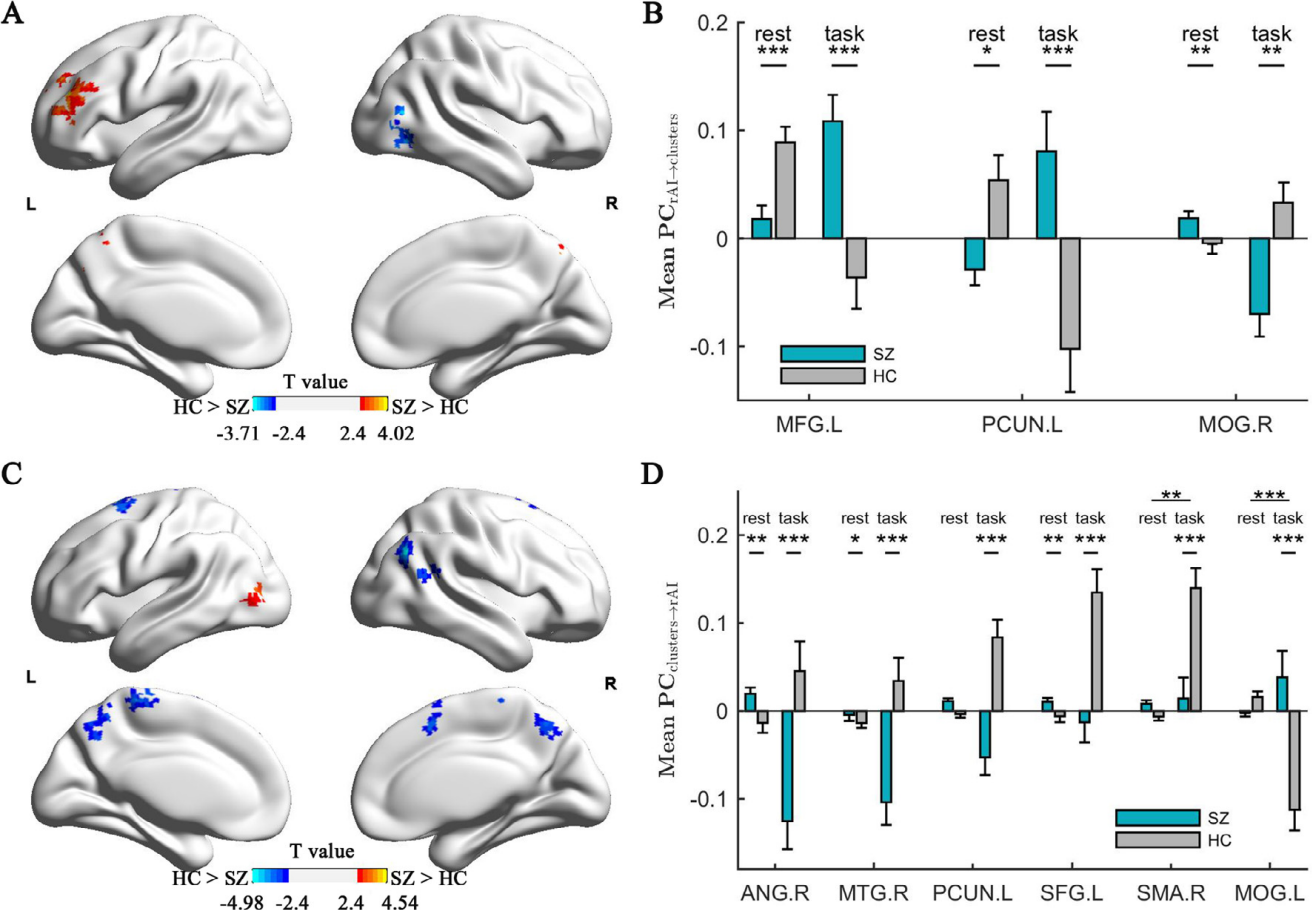
Group difference in the change of rAI-Brain interaction when shifting from rest-to-task between patients and controls. Change of path coefficient (CPC) at the identified clusters from rAI to whole brain (A) and from whole brain to rAI (C); Mean path coefficient (PC) of each cluster from rAI to whole brain (B) and from whole brain to rAI (D) (*p < 0.05, **p < 0.005, ***p < 0.0005).

Three clusters located at the left middle frontal gyrus (MFG) (*T*_54_ = 3.93, *p* = 2.4 × 10^−4^ at peak voxel), left precuneus (PCUN) (*T*_54_ = 3.86, *p* = 3.1 × 10^−4^ at peak voxel) and right middle occipital gyrus (MOG) (*T*_54_ = −3.59, *p* = 7.1 × 10^−4^ at peak voxel), exhibited significant differences in CPC between SZ and HC groups (Fig. 1A and Table 2). During task compared to resting state (rest-to-task contrast), the influence of rAI on the left MFG and left PCUN decreased in the healthy controls but increased in patients. In contrast, the influence of the rAI on the cluster at the right MOG decreased in patients, while it increased in controls when moving from rest-to-task state (Table S3). These findings cannot be simply explained by altered brain activation as none of these identified clusters was overlapping with those clusters having group-different changes of brain activation from rest to task (Table S2).

**Table 2:** The areas with significantly different CPC (from rAI) between SZ and HC groups.^a^

	Area	SZ	HC	*T*_54_	*p*	Cohen’s *d*	MNI			*K*
*SZ > HC*										
1	MFG.L	0.113 (0.29)	−0.179 (0.28)	3.93	2.4 × 10^−4^	1.04	−33	48	24	157
2	PCUN.L	0.175 (0.31)	−0.170 (0.32)	3.86	3.1 × 10^−4^	1.10	−3	−51	60	119

*SZ < HC*										
3	MOG.R	−0.107 (0.17)	0.062 (0.20)	−3.59	7.1 × 10^−4^	−0.90	42	−75	3	94

a The statistics at the peak voxel were listed. ‘K’ means the cluster size, ‘T’ is the t-statistic, ‘P’ is the corresponding p value, and ‘MNI’ is the Montreal Neurological Institute coordinates. The mean value and the standard deviation (SD, in brackets) were both listed in SZ and HC group.

For the identified three clusters, considering the averaged PC and CPC among all voxels within each cluster, we found the corresponding results became more pronounced (Cohen’s *d* = 1.35, *T*_54_ = 5.19, *p* = 2.8 × 10^−6^ from rAI to left MFG; *d* = 1.22, *T*_54_ = 4.72, *p* = 1.5 × 10^−5^ from rAI to left PCUN; *d* = −1.00, *T*_54_ = −3.88, *p* = 2.6 × 10^−4^ from rAI to right MOG), as the mean statistics had less variation than the peak (Fig. 1B and Table S3). After controlling for the brain activation in the seed region (i.e. the rAI), group differences of CPC in these identified clusters remained significant (Table S8).

### 3.2 Change in path coefficient to rAI

With rest-to-task contrast, the path coefficients from the visual cortex (left MOG) to rAI increased (*T*_54_ = 4.54, *p* = 3.2 × 10^−5^ at peak voxel) in patients with SZ compared to HC (Fig. 1C and Table 3). At this left MOG cluster, HC showed a decreased (*T_30_* = −4.80, p = 4.1 × 10^−5^) influence on rAI during task compared to rest, while patients showed a non-significant increase (*T*_28_ = 1.34, *p* = 0.1908) in influence from left MOG to rAI.

**Table 3:** Areas with significantly different CPC (to rAI) between SZ and HC groups.

	Area	SZ	HC	*T*_54_	*p*	Cohen’s *d*	MNI			*K*
1	MOG.L	0.047(0.23)	−0.19(0.18)	4.54	3.2×10^−5^	1.15	−36	−78	9	60

*SZ < HC*										
2	ANG.R	−0.166(0.17)	0.074(0.20)	−4.98	6.8×10^−6^	−1.05	48	−66	33	64

*SZ < HC*										
3	PCUN.L	−0.100(0.15)	0.131(0.20)	−4.80	1.3×10^−5^	−1.39	−3	−48	57	336
4	MTG.R	−0.102(0.15)	0.085(0.19)	−4.51	3.6×10^−5^	−1.01	57	−54	18	90
5	SFGdor.L	−0.038(0.17)	0.154(0.18)	−3.94	2.4×10^−4^	−1.20	−18	9	63	65
6	SMA.R	0.034(0.21)	0.213(0.17)	−3.57	7.6×10^−4^	−1.11	9	15	51	74

In 5 other clusters, patients manifested decreased [left PCUN (*T*_54_ = −5.31, *p* = 2.1 × 10^−6^), left SFGdor (*T*_54_ = −4.47, p = 4.0 × 10^−5^), right ANG (T_54_ = −4.07, *p* = 1.5 × 10^−4^), right MTG (*T*_54_ = −4.28, *p* = 7.8 × 10^−5^) and right SMA (*T*_54_ = −4.23, *p* = 9.0 × 10^−5^)] CPC compared to HC groups (Fig. 1D and Table 3). In HC, all of these clusters had an increase while patients had a decrease in influence on rAI during task compared to rest (Fig. 1D and Table S4). Except for the cluster in the left PCUN, other clusters did not overlap with those exhibiting significant group-difference in task-related activation (Tables 3 and S2).

### 3.3 Behaviour and symptomatic correlations of the CPC

**Fig. 2:**
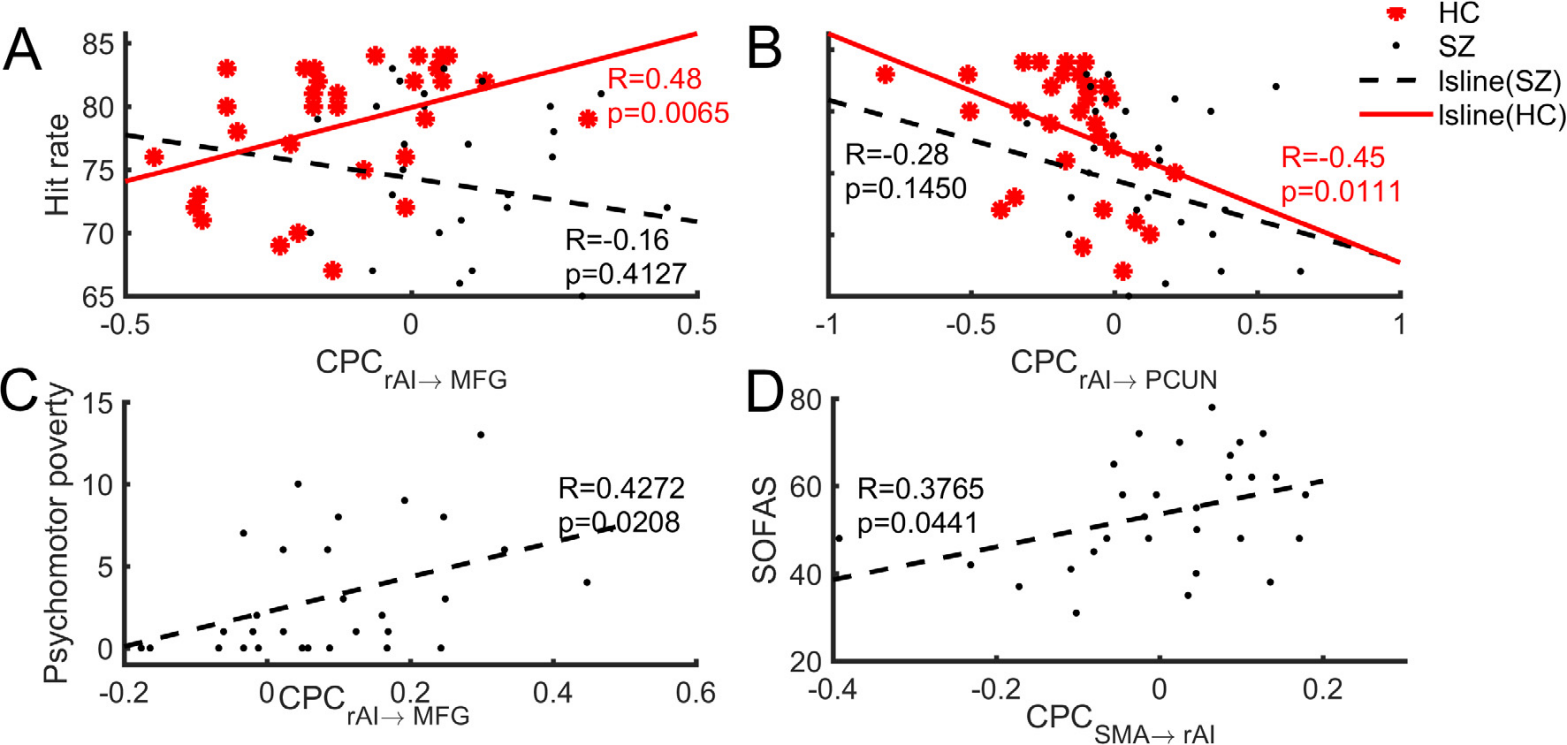
Behavior and symptomatic correlations of the CPC. For the hit rate of 2-back task, correlations are shown in (A) and (B) for healthy controls (red filled circles) and patients with schizophrenia (black dots). For the clinical symptoms in patients, correlations are shown in (C) Psychomotor Poverty and (D) SOFAS. (For interpretation of the references to colour in this figure legend, the reader is referred to the web version of this article.)

In healthy controls (Fig. 2A and B), we found the CPC from rAI to left MFG was positively associated (*r* = 0.478, *p* = 0.0065, uncorrected, *n* = 31) with task performance (the hit rate of 2-back task), while the CPC from rAI to left PCUN was negatively correlated (*r* = −0.45, *p* = 0.0111, uncorrected, *n* = 31) with the task performance. These associations were significant (*p* < 0.05, uncorrected, Table S5) even after adjusting for covariates (age, gender, two head motion parameters). However, both associations were absent in patients (Fig. 2A and B), and the correlation between CPC of rAI-to-left MFG and the hit rate was significantly greater in healthy controls than patients (z = 2.51, p = 0.0060, by one-tailed Fisher’s r-to-Z test). The correlation of CPC of rAI-to-left PCUN and the hit rate did not significantly differ between the groups (Fisher’s r-to-Z test p = 0.47; total sample r after controlling for the diagnosis = −0.3871, p = 0.0032).

In patients with schizophrenia (Fig. 2C and D), the increased CPC from rAI to left MFG correlated with higher psychomotor poverty (*r* = 0.427, *p* = 0.0208, *n* = 29). The reduced CPC from right SMA to rAI was associated with lower SOFAS (*r* = 0.3765, *p* = 0.0441, uncorrected, *n* = 29). There were no correlations between CPC values of any of the clusters and current or cumulative life time exposure to antipsychotics (all *p* ≥ 0.09, Tables S6 and S7).

### 3.4 The effect of brain activation, functional connectivity, task load and seed location on group differences in CPC

The group-difference in CPC remained significant even after controlling for the brain activation of the seed region and the identified cluster (see Supplement Table S8). We further demonstrated the specificity of our results to one-TR lag GCA of rAI by contrasting this with GCA of a posterior insula seed, as well as computing the change coefficient for functional connectivity of the rAI seed. We also confirmed that the change in effective connectivity of rAI to the MFG and PCUN nodes, as well as SMA to rAI were disrupted but less pronounced with the 1-back compared to the 2-back load effect (see Supplement Tables S9–S11).

## 4 Discussion

Though system-level aberrations in resting-state networks have been repeatedly shown in schizophrenia (Dong et al., 2018, Kambeitz et al., 2016, Woodward et al., 2011), to our knowledge this is the first study to directly contrast the effective connectivity of anterior insula between task-positive and task-negative states in schizophrenia. Using a directed connectivity approach, we first report an aberrant task-related increase in the influence of the rAI to MFG (Cohen’s *d* = 1.35) and precuneus (*d* = 1.22), alongside a task-related decrease in inflow into rAI from distributed brain regions in schizophrenia. In healthy controls, lower n-back performance was related to a task-related reduction in rAI-to-MFG but an increase in rAI-to-PCUN effective connectivity. However, despite significantly lower performance, patients had a non-selective increase in rAI’s influence on both MFG and PCUN. These observations confirm our primary hypothesis that control signals from rAI are abnormally elevated and directed towards both task-positive and task-negative brain regions, when task-related demands arise in schizophrenia. This aberrant surge in proximal salience (or SN signalling, inferred from Granger-causal dependencies) to DMN and CEN may indicate a system-level failure of resource allocation when cognitive demands arise (Sheffield et al., 2016), contributing to the working memory deficits that are robustly observed in schizophrenia (Park and Holzman, 1992, Yang et al., 2020). Thus, our results add to a growing body of evidence implicating a crucial role of rAI-centered triple network abnormality in schizophrenia (Palaniyappan et al., 2019, Supekar et al., 2019, Wotruba et al., 2013), and clarify an important link between the working memory and the triple network system.

To our knowledge, only one other fMRI study has investigated connectivity of the salience network changes in schizophrenia during a cognitive task *vs*. rest (Repovš and Barch, 2012). In line with Repovs and Barch, we also found evidence of decreased input of the DMN hubs (angular gyri, precuneus) to rAI. But Repovs and Barch also noted a task-related reduction in functional connectivity between SN (identified as cingulo-opercular network) and DMN, in contrast to our observation of an increase in SN (rAI) to DMN (precuneus) connectivity. It is important to note that Repovs and Barch studied non-directed functional connectivity and included both clinically healthy siblings and patients with schizophrenia in a single group in order to study the genetic liability. Furthermore, the evoked functional activation and connectivity from the 0-back is likely to differ from the 2-back task used in our study (Barch et al., 2013, Rottschy et al., 2012).

We note that the visual (dorsal and lateral occipital) cortex has a specific task-related divergence in effective connectivity to and from the rAI in schizophrenia. This task-related increase in MOG-to-rAI influence occurs in sharp contrast to the generalised reduction in influence from other brain regions to rAI in schizophrenia. Notably, this increase in MOG-to-rAI influence occurs in patients irrespective of their symptomatic and functional status, indicating a breakdown of a crucial bottom-up pathway that feeds sensory information to the salience network (Sterzer and Kleinschmidt, 2010). Mechanistic models that place salience network at the apex of hierarchical information-processing brain networks (Goulden et al., 2014, Menon and Uddin, 2010, Zhou et al., 2018), often focus primarily on the DMN, the CEN and the SN. Extant findings, including the earliest works identifying the distributed salience processing cortical system (Downar et al., 2002), emphasize the relevance of visual and other sensory inputs to the SN that likely sets up the contextual tone for coordinating the neuronal workspace (Mišić et al., 2015, Riedl et al., 2016). Our observation of a distinct pattern of abnormality in the visuo-salience circuitry indicates that the top-down and bottom-up pathways in the hierarchical processing system centered on the rAI are differentially affected in schizophrenia. This observation extends the notion of sensory processing deficits in schizophrenia, proposed by Javitt (2009) and raises the possibility that with the increased demand of 2-back processing, patients show an aberrant renunciation of bottom-up processing at the expense of top-down mode of salience signalling.

We noted higher task-related outflow signal from rAI-to-MFG (and lower rAI-to-PCUN) was associated with higher hit rate in healthy controls, indicating that differential signalling from rAI-to-DMN/CEN nodes may serve to dissociate the two competing information processing systems and thus increase the accuracy of stimulus–response association. This observation is in line with several studies that note that the functional divergence of DMN and CEN aids in task performance (Anticevic et al., 2012, Prado and Weissman, 2011, Sala-Llonch et al., 2012). On the other hand, the task-state evoked effective connectivity from rAI to both DMN and CEN nodes was elevated in our patient sample, with no associated gain in task performance. This observation bridges the notion of inefficient and inappropriately excessive recruitment of lateral prefrontal regions (CEN) proposed by Manoach and colleagues (2003), as well as an increase in midline cortical (DMN) engagement in schizophrenia during 2-back performance (Godwin et al., 2017, Guerrero-Pedraza et al., 2012).

We noted that the rAI-to-MFG outflow, but not the rAI-to-precuneus outflow, was pronounced in patients with a high degree of psychomotor poverty (negative symptoms), consistent with a prior report (Manoliu et al., 2013) using independent component analysis of between-network connectivity at rest. This supports the speculation that the bottom-up rAI-to-MFG guidance of action-decisions is inefficient (or intrusive), contributing to volitional deficits by disabling the task-positive system and presenting clinically as negative symptoms with psychomotor poverty. In a predictive coding framework, if the mismatch between higher-order expectations and incoming (bottom-up) information (i.e. an error signal) is ineffective in updating the expectations, this may lead to persistent uncertainty regarding future sensory experiences, ultimately leading to stimulus avoidance and withdrawal. These symptoms are clinically measured using SSPI as psychomotor poverty (Corlett et al., 2016). This conjecture can be directly tested in future studies, alongside competing models of reward learning and decision making (Deserno et al., 2017).

Our study has several strengths. Notably, we used an approach of effective connectivity that aids us to parse directionality of network connectivity. Further, by employing a distinct 10-minutes long resting session followed by the task paradigm, we avoided task-related spill-over effects on the resting data. There were several limitations as well in the current study. We studied a sample of medicated patients, which may limit our results since dopamine-blocking agents are known to affect connectivity patterns (Kraguljac et al., 2016, Nejad et al., 2012). Nevertheless, we did not find any linear relationship between cross-sectional or cumulative dose of antipsychotic exposure and the change in path coefficients of the reported clusters. Second, we used data collected at a single time point to study a neurophysiological ‘change’ phenomenon. Using more than one resting session would have allowed us to see if the observed rest-to-task changes revert to the resting state as expected in the two groups. Elton and Gao (2014) reported that task-related divergence in SN connectivity promptly reverts to the resting state in healthy controls when using a rest-task-rest design to examine this issue. Interpreting neural connectivity from Granger-causal models of fMRI data has certain limitations, discussed at length in previous works (for a review see Deshpande et al., 2011, Schippers et al., 2011, Seth et al., 2013, Smith et al., 2011). In particular, when two groups are compared, differences in Granger causal coefficients provide a meaningful measure of pathophysiology (Hamilton et al., 2011, Iwabuchi and Palaniyappan, 2017, Iwabuchi et al., 2014), even if the source of such differences is not fully known (Deshpande and Hu, 2012, Wen et al., 2013). Furthermore, the effective connectivity with other brain networks is well replicated, irrespective of the causal models employed to the fMRI time series data in both healthy controls (Goulden et al., 2014, Sridharan et al., 2008) and in patients with schizophrenia (Moran et al., 2013, Schmidt et al., 2016, Zhou et al., 2018). Given the lack of other task-fMRI experiments for these participants, we are unable to confirm if the apparent surge in rAI signalling for rest-to-task change is specific to working memory paradigms.

We conclude that the aberrant distribution of salience signals generated by the SN disrupts the discriminatory neural processes required for contextually relevant responses, providing a parsimonious explanation for the neurocognitive deficits that lie at the core of schizophrenia. Approaches that directly modulate the effective connectivity of anterior insula (Iwabuchi et al., 2017, Philip et al., 2018, Torres et al., 2016), could provide an experimental proof to this formulation. Furthermore, determining the trajectory of SN dysfunction from an asymptomatic stage to the chronic stages of schizophrenia could provide vital clues as to the factors that contribute to this aberration, as well as offering insights into the heterogeneity of cognitive outcomes in this illness.

## Funding

This work was supported by the Medical Research Council, UK (grant number G0601442). LP is supported by the Tanna Schulich Endowment Chair, the Academic Medical Organization of South-Western Ontario (Opportunities Fund) and the 10.13039/100009007Canadian Institutes of Health Research Foundation Grant (grant number 375104). QL was supported by the 10.13039/501100001809National Natural Science Foundation of China (grant numbers 81873909, 81930095, and 81761128011), Shanghai Municipal Science and Technology Major Project (grant number 2018SHZDZX01), and Zhangjiang Lab. HG was supported by the 10.13039/501100001809National Natural Science Foundation of China (grant numbers 11872276 and 11572225). PFL receives research funding from NIHR Nottingham Biomedical Research Centre.

Disclosures

Dr Palaniyappan reports speaker fees from Janssen and Otsuka Canada, Canadian Psychiatric Association and SPMM Course (UK); investigator-initiated educational grants from Janssen Canada, Otsuka Canada and Sunovion not related to the submitted work. The other authors reported no biomedical financial interests or potential conflicts of interest.

## Contributions

L.P. and P.L. conceptualized the study. Q.L and L.P. designed the methodology. Q.L., B.P., H.G., and L.P performed most of the analyses. M.S., S.F., P.L. and L.P. acquired and processed neuroimaging, behavioural and clinical data. Q.L., B.P., and L.P. wrote the manuscript. H.G., M.S., S.F., and P.L made critical revision of the manuscript. P.L., L.P. and Q.L. acquired the funding. All authors reviewed the manuscript and discussed the work.
