# Supplementary material for: Effective connectivity of the right anterior insula in schizophrenia: The salience network and task-negative to task-positive transition
Source: Neuroimage Clin. 2020 Aug 7;28:102377. doi: 10.1016/j.nicl.2020.102377 (PMC7451428; doi:10.1016/j.nicl.2020.102377)
Supplement: Supplementary data 1 [file mmc1.docx]

**Supplemental Information**

Effective connectivity of the right anterior insula in schizophrenia: the salience network and task-negative to task-positive transition

Q Luo, B Pan, H Gu, *et al*.

Table of Contents

[Figure S1: N-back working memory task. 1](#_Toc45983386)

[Figure S2: Overall design of the study 2](#_Toc45983387)

[Figure S3. T maps of CPC in both HC and SCZ groups and the direction influence 3](#_Toc45983388)

[Method S1: Correcting for head movements 4](#_Toc45983389)

[Table S1. Clinical scores of patients with SZ (n=29). 5](#_Toc45983390)

[Table S2. Group comparison of brain activation during 2-back task between patients and controls. 6](#_Toc45983391)

[Table S3. Path coefficients from rAI to various clusters in patients and controls for rest-to-task contrast. 7](#_Toc45983392)

[Table S4. Path coefficients from various clusters to rAI in patients and controls shifting from rest-to-task. 8](#_Toc45983393)

[Table S5. Behavioral association conditioned on covariates. 9](#_Toc45983394)

[Table S6. Medications data. 10](#_Toc45983395)

[Table S7. Significance level (p-value) of the correlation between antipsychotic exposure and mean CPC of the identified clusters. 11](#_Toc45983396)

[Table S8. Diagnostic differences in CPC (to and from rAI) are independent of the degree of task-related activation of the seed (rAI). 12](#_Toc45983397)

[Table S9. Group comparison of the functional connectivity between the seed region, rAI, and the significant clusters identified in the main text. 13](#_Toc45983398)

[Table S10. Group comparison of the CPC between the right posterior insula (rPI) and the 11 significant clusters. 14](#_Toc45983399)

[Table S11. Group comparison of the CPC of the rest-to-1back change between the rAI and the 11 significant clusters. 15](#_Toc45983400)

[Table S12. Significant clusters of CPC from rAI to whole-brain in HC group. 16](#_Toc45983401)

[Table S13. Significant clusters of CPC from whole-brain to rAI in HC group. 17](#_Toc45983402)

[Table S14. Significant clusters of CPC from rAI to whole-brain in SCZ group. 18](#_Toc45983403)

[Table S15. Significant clusters of CPC from whole-brain to rAI in SCZ group. 19](#_Toc45983404)

**Figure S1: N-back working memory task.**

Seven task blocks each of 110s duration were presented in each session. Each task block consisted of 0-back, 1-back, and 2-back conditions of 30s duration each presented in a random sequence, with 10s interval between the conditions. On-screen instructions preceded every condition indicating the type of response required (0-, 1-, or 2-back; 2 seconds). Each condition included 4 target and 11 non-target stimuli with a 2s inter-stimulus interval.

**Figure S2: Overall design of the study**

PC: Path Coefficient, CPC: Change in path coefficient (rest-minus-task), rAI – right anterior insula

**Figure S3. T maps of CPC in both HC and SCZ groups and the direction influence**

**
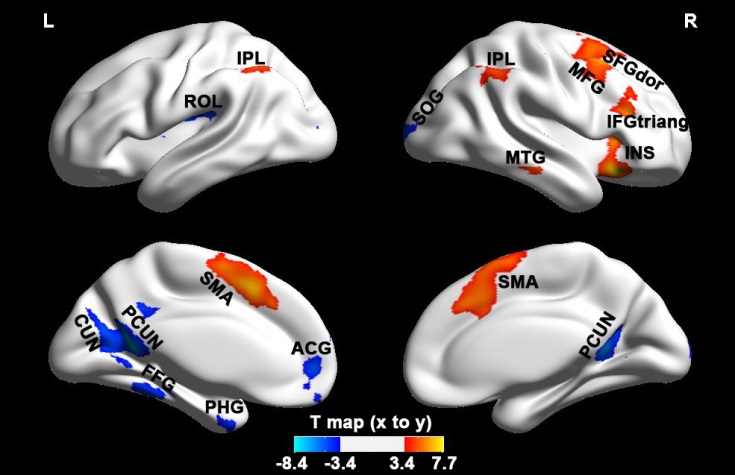

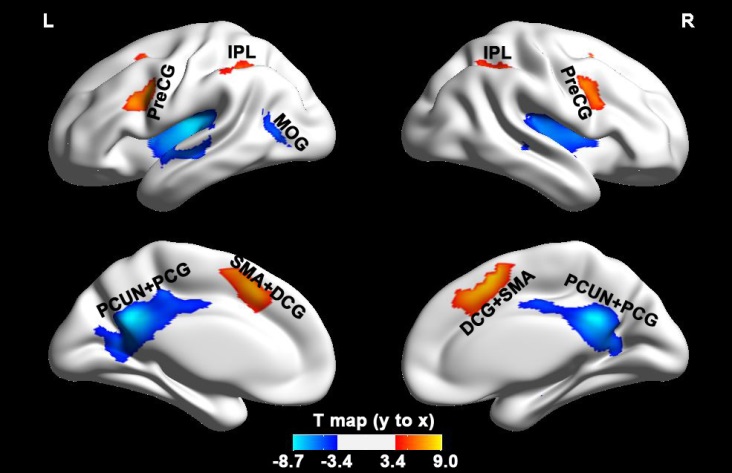
**

(a) from rAI to whole-brain, HC group; (b) from whole-brain to rAI, HC group;


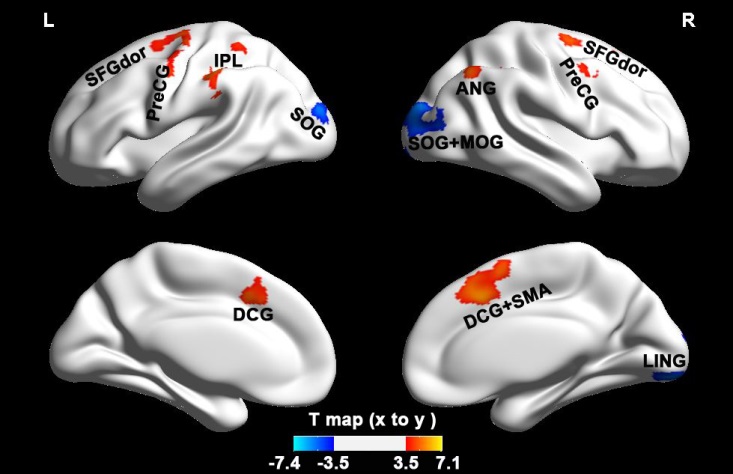

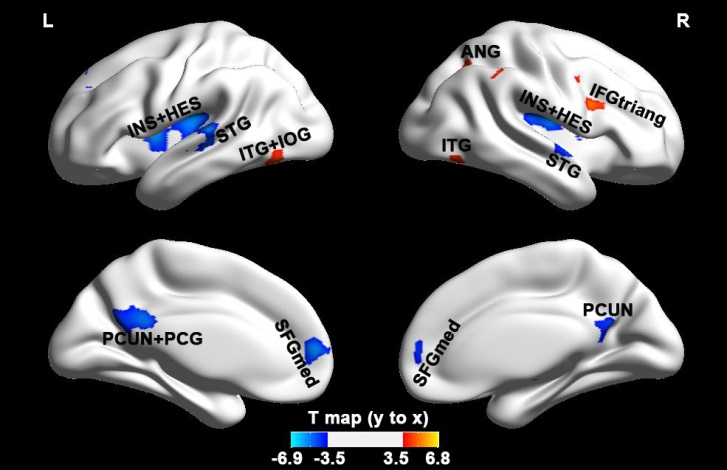


(c) from rAI to whole-brain, SCZ group; (d) from whole-brain to rAI, SCZ group.

We calculated the change of the path coefficient (CPC) from resting to task, and assessed its significance by one-sample t test. We conducted this seed-based analysis in both HC and SCZ separately, and the significant CPC was identified after the Alpha-Sim (Monte Carlo simulations) correction for multiple comparisons with voxel-level p value < 0.001, cluster p value < 0.05.

**Method S1: Correcting for head movements**

In addition to the precautions taken during image acquisition, several other measures were employed to control for movement-induced artifacts. Firstly, motion parameters in three planes were assessed for each participant, and participants with movement >3 mm or 3^0^ were excluded from the analysis. Secondly, displaced frames (defined as frames with summed displacement across all six rigid body motion parameters exceeding 0.5mm or root-mean-square of volume signal intensity difference that exceeded ±3 standard deviation of the average across all scans), along with 1 preceding and 2 succeeding frames were replaced using a nearest (unaffected) neighbour interpolation method using ArtRepair software (http://cibsr.stanford.edu/tools/human-brain-project/artrepair-software.html). Thirdly, to remove the variance in connectivity spuriously introduced by head motion, we used the head motion parameters as nuisance covariates when extracting the timeseries of BOLD signals. Fourth, we compared the overall frame-wise displacement levels between the two groups to detect the possibility of systematic differences in head motion in line with Power et al. (2012). Fifth, we regressed out mean frame-wise displacement from the CPC before undertaking group comparison. Finally, we tested all behavioral and clinical associations after linear adjustment for the mean frame-wise displacement to detect any confounding role of head motion in our results.

No significant difference was noted in the total proportion of frames with displacement (proportion of displaced frames (SD) in subjects with schizophrenia =4.4%(10.1%); controls=1.9%(3.6%); t(55)=1.16, p=0.2494) and for the mean displacement across the 3 translation and 3 rotation axes, quantified in accordance with Power et al. (2012) (framewise displacement (SD) in subjects with schizophrenia = 0.070 (0.041); controls = 0.061 (0.030); t(55)= 0.9890, p=0.3270).

We correlated the mean frame-wise displacement (Power et al., 2012) to the CPC of MFG.L, PCUN.L and MOG.R in each group separately, using an uncorrected threshold of p=0.05 to flag any suspected relationships. There were no significant correlations within the sample (all p>0.0586). This strengthens the claim that the observed patients vs. healthy controls differences are due to changes in brain connectivity, rather than putative motion induced relationships.

**Table S1. Clinical scores of patients with SZ (n=29).**

| Scale | Symptoms | Mean (SD) |  |
| --- | --- | --- | --- |
| continuous | Mean SOFAS score | 53.8 (12.7) |  |
|  | Mean total SSPI score | 12.5 (7.4) |  |
| discrete | Reality Distortion | 2.3 (2.6) |  |
|  | Psychomotor Poverty | 3.2 (3.7) |  |
|  | Disorganization | 1.4 (1.4) |  |

**Table S2. Group comparison of brain activation during 2-back task between patients and controls.**

|  | **Area** | **T_54_** | **P** | **MNI** | | | **K** |
| --- | --- | --- | --- | --- | --- | --- | --- |
| **SZ > HC** | | | | | | | |
| **1** | DCG | 3.48 | 1.0×10^-3^ | -6 | -9 | 30 | 114 |
| **2** | INS.R | 3.79 | 3.8×10^-4^ | 39 | 6 | 3 | 83 |
| **SZ < HC** | | | | | | | |
| **3** | PCUN | -3.90 | 2.7×10^-4^ | -3 | -60 | 42 | 272 |

To see the significance level of the group difference at the insula, the threshold of cluster size was set to be 80, although the actual threshold given by the Alpha-Sim correction was 127. ‘K’ is the cluster size, ‘T’ is the t-statistic of two-sample t test, ‘P’ is the corresponding p-value, and ‘MNI’ is the Montreal Neurological Institute coordinates. In order to detect group difference of brain activation during 2-back task between patients and controls, we first calculated contrast map on brain mask (2-back task condition vs. rest condition) by SPM8, than using two samples t-test to detect group difference between patients and controls (controlled age, gender and head motion as covariance), a T map on brain mask was produced, then corrected by Alpha-Sim by setting the threshold of p value to 0.01(one-tailed) at voxel level, 0.05(one-tailed) at cluster value, so the threshold of significant cluster size was 127.

**Table S3. Path coefficients from rAI to various clusters in patients and controls for rest-to-task contrast.**

|  |  | **SZ** | **HC** | **SZ v.s. HC** |  |
| --- | --- | --- | --- | --- | --- |
| **MFG.L** | Rest-to-Task | 0.091 (0.002) | -0.125 (4.0×10^-4^) | 4.92(8.4×10^-6^) | |
|  | Resting | 0.018 (0.163) | 0.089 (1.1×10^-6^) | -3.78(4.0×10^-4^) | |
|  | Task | 0.108 (0.0001) | -0.036 (0.222) | 3.51(9.0×10^-4^) | |
| **PCUN.L** | Rest-to-Task | 0.11 (0.014) | -0.156 (2.5×10^-4^) | 4.46(4.2×10^-5^) | |
|  | Resting | -0.029 (0.055) | 0.054 (0.028) | -2.94(4.9×10^-3^) | |
|  | Task | 0.081 (0.036) | -0.102 (0.016) | 3.06(3.5×10^-3^) | |
| **MOG.R** | Rest-to-Task | -0.089 (0.001) | 0.038 (0.110) | -4.02(1.8×10^-4^) | |
|  | Resting | 0.018 (0.008) | -0.005 (0.569) | 2.23(3.0×10^-2^) | |
|  | Task | -0.07 (0.002) | 0.033 (0.087) | -3.78(4.0×10^-4^) | |

The mean path coefficient (PC) in the significant cluster was listed for both patients (SZ) and healthy controls (HC), and the significance level of such coefficient was assessed by the one-sample t-test (p-value was reported in brackets, degree-of-freedom was 28 for SZ and 30 for HC). The significance (p value) of the difference in the PC between patients and controls was given by two-sample t test (SZ vs. HC, degree-of-freedom was 54).

**Table S4. Path coefficients from various clusters to rAI in patients and controls shifting from rest-to-task.**

|  |  | **SZ** | **HC** | **SZ v.s. HC** |
| --- | --- | --- | --- | --- |
| **MOG.L** | Rest-to-Task | 0.041 (0.191) | -0.128 (4.1×10^-5^) | 4.24(8.8×10^-5^) |
|  | Resting | -0.003 (0.510) | 0.016 (0.020) | -2.28(2.7×10^-2^) |
|  | Task | 0.038 (0.215) | -0.112 (4.2×10^-5^) | 4.02(2.0×10^-4^) |
| **ANG.R** | Rest-to-Task | -0.145 (1.0×10^-4^) | 0.059 (0.135) | -4.07(1.5×10^-4^) |
|  | Resting | 0.019 (0.011) | -0.014 (0.237) | 2.75(8.1×10^-3^) |
|  | Task | -0.125 (0.001) | 0.045 (0.192) | -3.58(7.0×10^-4^) |
| **PCUN.L** | Rest-to-Task | -0.064 (0.003) | 0.087 (2.0×10^-4^) | -5.31(2.1×10^-6^) |
|  | Resting | 0.011 (0.002) | -0.004 (0.33) | 2.98(4.3×10^-3^) |
|  | Task | -0.053 (0.014) | 0.084 (2.0×10^-4^) | -4.76(1.5×10^-5^) |
| **MTG.R** | Task - rest | -0.099 (0.001) | 0.048 (0.09) | -4.28(7.8×10^-5^) |
|  | Resting | -0.005 (0.51) | -0.014 (0.02) | 1.08(0.29) |
|  | Task | -0.104 (4.0×10^-4^) | 0.034 (0.21) | -4.08(1.0×10^-4^) |
| **SFGdor.L** | Task - rest | -0.023 (0.326) | 0.141 (8.9×10^-6^) | -4.47(4.0×10^-5^) |
|  | Resting | 0.010 (0.031) | -0.006 (0.38) | 1.83(7.3×10^-2^) |
|  | Task | -0.013 (0.578) | 0.135 (1.7×10^-5^) | -4.02(2.0×10^-4^) |
| **SMA.R** | Task - rest | 0.006 (0.798) | 0.146 (4.2×10^-7^) | -4.23(9.0×10^-5^) |
|  | Resting | 0.008 (0.047) | -0.006 (0.16) | 2.44(1.8×10^-2^) |
|  | Task | 0.014 (0.552) | 0.140 (9.1×10^-7^) | -3.76(4.0×10^-4^) |

**Table S5. Behavioral association conditioned on covariates.**

Correlation coefficients are listed, and the corresponding p-values are reported in brackets.

|  | **Co-variates** | **Degree**  **of**  **freedom** | **Effective connectivity** | |
| --- | --- | --- | --- | --- |
|  |  |  | **rAI 🡪 MFG.L** | **rAI 🡪PCUN.L** |
| **Hit rate *controls*** | Age&Sex | 27 | 0.45(0.0133) | -0.44(0.0170) |
|  | Translation&Rotation | 27 | 0.39(0.0341) | -0.45(0.0147) |
|  |  |  | **rAI 🡪 MFG.L** |  |
| **PM Poverty** ***patients*** | Age&Sex | 25 | 0.38(0.0533) |  |
|  | Translation&Rotation | 25 | 0.41(0.0359) |  |
|  | DOI&CPZ&Cumulative | 19 | 0.48(0.0276) |  |
|  |  |  | **SMA.R🡪rAI** |  |
| **SOFAS *patients*** | Age&Sex | 25 | 0.35(0.0729) |  |
|  | Translation&Rotation | 25 | 0.37(0.0516) |  |
|  | DOI&CPZ&Cumulative | 19 | 0.33(0.1482) |  |

**Table S6. Medication data.**

Medication related information from the original recruited sample reported in Palaniyappan & Liddle 2013 (Palaniyappan and Liddle, 2013); this includes patients whose imaging data were excluded due to artifacts or quality issues for the current study)

| **Psychotropic medication** | **Number of patients** |
| --- | --- |
| Amisulpride | 1 |
| Aripiprazole | 5 |
| Chlorpromazine | 1 |
| Citalopram | 3 |
| Clozapine | 6 |
| Fluoxetine | 4 |
| Fluphenazine depot | 1 |
| Mirtazapine | 1 |
| Olanzapine | 9 |
| Piportil depot | 1 |
| Quetiapine | 2 |
| Risperidone depot | 5 |
| Risperidone oral | 7 |
| Sertraline | 1 |
| Sodium Valproate | 3 |
| Venlafaxine | 2 |
| Zuclopenthixol depot | 1 |
| Unmedicated >3 months | 4 |

| ROIs | | **DOI** (n = 29) | | **CPZ** (n = 24) | | **Cumulative** (n = 24) | |
| --- | --- | --- | --- | --- | --- | --- | --- |
|  |  | r | p | r | p | r | p |
| **from-rAI** | MFG.L | -0.32 | 0.0898 | -0.24 | 0.2512 | -0.16 | 0.4583 |
|  | PCUN.L | -0.14 | 0.4841 | 0.28 | 0.1803 | 0.34 | 0.104 |
|  | MOG.R | -0.13 | 0.5008 | -0.17 | 0.4405 | -0.29 | 0.167 |
| **to-rAI** | ANG.R | -0.19 | 0.3151 | 0.03 | 0.8787 | -0.01 | 0.9477 |
|  | MTG.R | -0.14 | 0.4542 | -0.17 | 0.4204 | -0.08 | 0.7107 |
|  | PCUN.L | -0.15 | 0.4405 | 0.08 | 0.7193 | 0.02 | 0.9402 |
|  | SFG.L | -0.16 | 0.3966 | 0.11 | 0.6036 | -0.04 | 0.865 |
|  | SMA.R | -0.22 | 0.2429 | 0.05 | 0.8062 | -0.13 | 0.5575 |
|  | MOG.L | 0.08 | 0.6718 | 0.32 | 0.128 | 0.15 | 0.4819 |

**Table S7. Significance level (p-value) of the correlation between antipsychotic exposure and mean CPC of the identified clusters.**

**Table S8. Diagnostic differences in CPC (to and from rAI) are independent of the degree of task-related activation of the seed (rAI).**

| ROIs | | **SZ vs. HC** | **SZ vs. HC (controlled for seed activation)** | |
| --- | --- | --- | --- | --- |
|  |  | T_54_ (p value) | | T_53_ (p value) |
| **from-rAI** | MFG.L | 4.92 (8.4×10^-6^) | | 4.72 (8.4×10^-6^) |
|  | PCUN.L | 4.46 (4.2×10^-5^) | | 4.42 (5.0×10^-5^) |
|  | MOG.R | -4.02 (1.8×10^-4^) | | -4.11 (1.4×10^-4^) |
| **to-rAI** | ANG.R | -4.07 (1.5×10^-4^) | | -4.04 (1.7×10^-4^) |
|  | MTG.R | -4.28 (7.8×10^-5^) | | -4.32 (6.7×10^-5^) |
|  | PCUN.L | -5.31 (2.1×10^-6^) | | -4.62 (2.5×10^-5^) ^c^ |
|  | SFG.L | -4.47 (4.0×10^-5^) | | -4.39 (5.4×10^-5^) |
|  | SMA.R | -4.23 (9.0×10^-5^) | | -4.03 (1.8×10^-4^) |
|  | MOG.L | 4.24 (8.8×10^-5^) | | 4.05 (1.7×10^-4^) |

Because the cluster PCUN.L, which was shown group difference on CPC between SZ and HC groups, have overlap voxels (89 voxels) with the area in Table S2, which were shown group difference on activation, in order to control the confound influence on group difference on CPC, the activation of cluster PCUN.L was also controlled as covariance. So, the degree of freedom of the T statistic in this case was 52.

**Table S9.** **Group comparison of the functional connectivity between the seed region, rAI, and the significant clusters identified in the main text.**

| **Clusters defined by CPC** | **ROI** | **T statistics of FC differences**  **(rest FC vs. task FC)** | **p-value** |
| --- | --- | --- | --- |
| From rAI | MFG_L | 0.87 | 0.39 |
|  | MOG_R | -1.01 | 0.32 |
|  | PCUN_L | 0.84 | 0.40 |
| To rAI | ANG_R | 0.09 | 0.93 |
|  | MOG_L | -0.88 | 0.38 |
|  | MTG_R | -0.26 | 0.79 |
|  | PCUN_L | -0.92 | 0.36 |
|  | SFG_L | -1.51 | 0.14 |
|  | SMA_R | 1.30 | 0.20 |

To test whether the patient vs. control differences in CPC maps were specific for the effective connectivity based on GCA, we assessed the corresponding group difference based on functional connectivity (FC) between the same seed region and clusters reported to be significant in the primary analysis of GC-based CPC. We found that the group differences of the corresponding FC were not significant (Table S9), suggesting that the findings based on GCA analysis do not arise from differences in functional connectivity, but are distinct deficits in the effective connectivity involving the rAI in schizophrenia.

**Table S10. Group comparison of the CPC between the right posterior insula (rPI) and** **the 11 significant clusters.**

| **Direction** | **ROI** | **CPC** | **p-value** | **fdr-p** |
| --- | --- | --- | --- | --- |
| **From rPI** | MFG_L | 1.79 | 0.08 | 0.29 |
|  | MOG_R | -2.42 | 0.02 | 0.17 |
|  | PCUN_L | -0.58 | 0.57 | 0.85 |
| **To rPI** | ANG_R | 1.49 | 0.14 | 0.29 |
|  | MOG_L | 1.61 | 0.11 | 0.29 |
|  | MTG_R | 0.08 | 0.94 | 0.94 |
|  | PCUN_L | -1.43 | 0.16 | 0.29 |
|  | SFG_L | -0.09 | 0.92 | 0.94 |
|  | SMA_R | 0.07 | 0.94 | 0.94 |

CPC: change of the path coefficient from rest to task;

ROI: region of interest, which was defined in the main text as the significant CPC clusters for rAI;

fdr-p: p-value corrected by the false discovery rate.

These significant clusters were identified in the main text for the right anterior insula. To test whether the patient vs. control differences in CPC maps were specific for rAI seed, we assessed the corresponding group difference based on GCA to and from a different seed region [the posterior insula (rPI: x=38, y=-4, z=10)]. We found that there were no group differences between patients and controls for the CPC maps involving rPI (Table S10), suggesting a distinct effective connectivity deficit involving rest vs. task states, pertaining to the right anterior insula seed of the salience network in schizophrenia.

**Table S11. Group comparison of the CPC of the rest-to-1back change between the rAI and the 11 significant clusters.**

| **Direction** | **ROI** | **rest-to-1back** | | | **rest-to-2back** | | |
| --- | --- | --- | --- | --- | --- | --- | --- |
|  |  | **CPC** | **p-value** | **fdr-p** | **CPC** | **p-value** | **fdr-p** |
| **From rAI** | MFG_L | 2.89 | 0.01 | 0.04 | 4.89 | 9.03×10^-6^ | 4.58×10^-5^ |
|  | MOG_R | -2.29 | 0.03 | 0.06 | -4.30 | 7.10×10^-5^ | 2.13×10^-4^ |
|  | PCUN_L | 2.57 | 0.01 | 0.04 | 3.67 | 5.58×10^-4^ | 1.00×10^-3^ |
| **To rAI** | ANG_R | -0.61 | 0.54 | 0.54 | -3.68 | 5.38×10^-4^ | 1.00×10^-3^ |
|  | MOG_L | 1.3 | 0.2 | 0.25 | 2.52 | 1.45×10^-2^ | 1.45×10^-2^ |
|  | MTG_R | -1.14 | 0.26 | 0.29 | -3.31 | 1.67×10^-3^ | 2.43×10^-3^ |
|  | PCUN_L | -1.31 | 0.2 | 0.25 | -4.86 | 1.02×10^-5^ | 4.58×10^-5^ |
|  | SFG_L | -2.03 | 0.05 | 0.08 | -2.92 | 5.03×10^-3^ | 5.66×10^-3^ |
|  | SMA_R | -2.7 | 0.01 | 0.04 | -3.26 | 1.89×10^-3^ | 2.43×10^-3^ |

These significant clusters were identified from the primary analysis reporting the rest-to-2back changes. To investigate the possibility of n-back load specific effects, we extracted the GCA maps during 1-back condition, and found a similar pattern of the group difference in the CPC but with a smaller effect size compared with the rest-to-2back shift. Compared with healthy controls, we found an increased CPC in both rAI🡪MFG_L and rAI🡪PCUN_L, and a decreased CPC in SMA_R🡪rAI in the patients with schizophrenia. After the FDR correction for multiple comparisons, all three group differences had a corrected p-value as 0.04 (Table S11). These results indicate that disrupted effective connectivity from rAI to DMN/CEN nodes as well as the reciprocal influence from SMA to the rAI worsens with the task difficulty in schizophrenia. This lends support to our view that an aberrant task-related surge in salience signalling from the rAI disrupts cognitive performance in schizophrenia.

**Table S12.** **Significant clusters of CPC from rAI to whole-brain in HC group.**

| **Area of peak** | **Peak MNI** | | | **PC of Rest (SD)** | **PC of Task (SD)** | **T** | **K** |
| --- | --- | --- | --- | --- | --- | --- | --- |
| INS.R | 33 | 21 | -15 | -0.004(0.07) | 0.129(0.10) | 7.66 | 1888 |
| IPL.L | -48 | -51 | 57 | 0.027(0.05) | 0.082(0.07) | 6.13 | 313 |
| MTG.R | 63 | -21 | -12 | -0.005(0.05) | 0.037(0.04) | 5.70 | 78 |
| IPL.R | 51 | -45 | 42 | 0.043(0.05) | 0.132(0.10) | 4.81 | 205 |
| PCUN.L | -15 | -39 | 24 | 0.001(0.04) | -0.066(0.04) | -8.40 | 909 |
| PCUN.R | 15 | -45 | 15 | 0.000(0.04) | -0.061(0.04) | -7.40 | 245 |
| ACG.L | -9 | 51 | 0 | -0.001(0.07) | -0.130(0.12) | -5.27 | 146 |
| CUN.R | 18 | -93 | 9 | 0.011(0.08) | -0.075(0.09) | -4.81 | 163 |
| PHG.L | -24 | 6 | -33 | 0.016(0.07) | -0.093(0.12) | -4.61 | 151 |

INS-- insula; IPL-- inferior parietal gyrus; MTG-- middle temporal gyrus; PCUN-- precuneus; ACG-- anterior cingulate; PHG-- parahippocampal gyrus; SD—standard deviation; PC—path coefficient; K—cluster size (i.e. number of voxels); L-- left hemisphere; R—right hemisphere.

**Table S13.** **Significant clusters of CPC from whole-brain to rAI in HC group.**

| **Area** | **Peak MNI** | | | **PC of Rest (SD)** | **PC of Task (SD)** | **T** | **K** |
| --- | --- | --- | --- | --- | --- | --- | --- |
| SMA.L | 0 | 18 | 45 | 0.003(0.02) | 0.109(0.07) | 8.97 | 1262 |
| IPL.R | 45 | -42 | 39 | -0.018(0.06) | 0.106(0.07) | 8.39 | 284 |
| IPL.L | -45 | -42 | 42 | -0.014(0.04) | 0.084(0.07) | 7.65 | 258 |
| PCG.L | -6 | -51 | 27 | -0.006(0.03) | -0.094(0.06) | -8.74 | 1595 |
| INS.L | -39 | -15 | 12 | 0.014(0.04) | -0.124(0.09) | -8.53 | 1600 |
| STG.R | 51 | -3 | 0 | 0.007(0.03) | -0.081(0.08) | -6.88 | 768 |

SMA—supplementary motor area; IPL-- inferior parietal gyrus; PCG-- posterior cingulate gyrus; INS-- insula; STG—superior temporal gyrus; SD—standard deviation; PC—path coefficient; K—cluster size (i.e. number of voxels); L-- left hemisphere; R—right hemisphere.

**Table S14. Significant clusters of CPC from rAI to whole-brain in SCZ group.**

| **Area** | **Peak MNI** | | | **PC of Rest (SD)** | **PC of Task (SD)** | **T** | **K** |
| --- | --- | --- | --- | --- | --- | --- | --- |
| IPL.L | -51 | -30 | 36 | -0.014(0.07) | 0.061(0.06) | 7.08 | 340 |
| DCG.L | 0 | 3 | 45 | -0.030(0.10) | 0.069(0.09) | 5.91 | 709 |
| ANG.R | 45 | -60 | 42 | 0.003(0.07) | 0.090(0.08) | 5.28 | 91 |
| PreCG.L | -33 | -12 | 60 | -0.004(0.04) | 0.075(0.07) | 4.91 | 219 |
| SOG.R | 21 | -87 | 18 | 0.024(0.05) | -0.071(0.06) | -7.42 | 806 |
| MOG.L | -24 | -87 | 18 | 0.025(0.05) | -0.052(0.06) | -6.29 | 130 |

IPL-- inferior parietal gyrus; DCG-- median cingulate gyrus; ANG-- angular gyrus; PreCG-- precental gyrus; SOG-- superior occipital gyrus; MOG—middle occipital gyrus; SD—standard deviation; PC—path coefficient; K—cluster size (i.e. number of voxels); L-- left hemisphere; R—right hemisphere.

**Table S15. Significant clusters of CPC from whole-brain to rAI in SCZ group.**

| **Area** | **Peak MNI** | | | **PC of Rest (SD)** | **PC of Task (SD)** | **T** | **K** |
| --- | --- | --- | --- | --- | --- | --- | --- |
| IPL.L | -27 | -57 | 42 | -0.013(0.04) | 0.104(0.09) | 6.81 | 179 |
| ANG.R | 30 | -63 | 45 | -0.006(0.03) | 0.074(0.07) | 5.78 | 213 |
| IOG.L | -48 | -69 | -12 | 0.000(0.04) | 0.071(0.06) | 5.74 | 252 |
| IFGoperc.R | 45 | 12 | 24 | -0.013(0.03) | 0.056(0.07) | 5.17 | 71 |
| ITG.R | 45 | -66 | -9 | -0.006(0.04) | 0.060(0.09) | 4.22 | 61 |
| INS.L | -39 | -18 | 24 | 0.005(0.04) | -0.100(0.08) | -6.85 | 1083 |
| SFGmed.L | -6 | 54 | 9 | -0.010(0.02) | -0.061(0.05) | -5.77 | 212 |
| STG.R | 51 | -18 | 6 | 0.006(0.03) | -0.057(0.06) | -5.60 | 381 |
| PCG.L | -6 | -42 | 24 | -0.004(0.03) | -0.084(0.08) | -5.21 | 352 |

IPL-- inferior parietal gyrus; ANG-- angular gyrus; IOG-- inferior occipital gyrus; IFGoperc-- Inferior frontal gyrus, opercular part.; ITG-- inferior temporal gyrus; INS-- insula; SFGmed-- Superior frontal gyrus, medial; STG-- superior temporal gyrus; PCG-- posterior cingulate gyrus; SD—standard deviation; PC—path coefficient; K—cluster size (i.e. number of voxels); L-- left hemisphere; R—right hemisphere.
